# Diarrhoea Complicating Severe Acute Malnutrition in Kenyan Children: A Prospective Descriptive Study of Risk Factors and Outcome

**DOI:** 10.1371/journal.pone.0038321

**Published:** 2012-06-04

**Authors:** Alison Talbert, Nahashon Thuo, Japhet Karisa, Charles Chesaro, Eric Ohuma, James Ignas, James A. Berkley, Christopher Toromo, Sarah Atkinson, Kathryn Maitland

**Affiliations:** 1 Kenya Medical Research Institute Wellcome Trust Research Programme, Kilifi, Kenya; 2 Centre for Clinical Vaccinology and Tropical Medicine, University of Oxford, Oxford, United Kingdom; 3 Department of Paediatrics, University of Oxford, Oxford, United Kingdom; 4 Wellcome Trust Centre for Clinical Tropical Medicine, Faculty of Medicine, Imperial College, Norfolk Place, London, United Kingdom; Aga Khan University, Pakistan

## Abstract

**Background:**

Severe acute malnutrition (SAM) accounts for two million deaths worldwide annually. In those hospitalised with SAM, concomitant infections and diarrhoea are frequent complications resulting in adverse outcome. We examined the clinical and laboratory features on admission and outcome of children with SAM and diarrhoea at a Kenyan district hospital.

**Methods:**

A 4-year prospective descriptive study involving 1,206 children aged 6 months to 12 years, hospitalized with SAM and managed in accordance with WHO guidelines. Data on clinical features, haematological, biochemical and microbiological findings for children with diarrhoea (≥3 watery stools/day) were systematically collected and analyzed to identify risk factors associated with poor outcome.

**Results:**

At admission 592 children (49%) had diarrhoea of which 122 (21%) died compared to 72/614 (12%) deaths in those without diarrhoea at admission (Χ^2^ = 17.6 p<0.001). A further 187 (16%) children developed diarrhoea after 48 hours of admission and 33 died (18%). Any diarrhoea during admission resulted in a significantly higher mortality 161/852 (19%) than those uncomplicated by diarrhoea 33/351 (9%) (Χ^2^ = 16.6 p<0.001). Features associated with a fatal outcome in children presenting with diarrhoea included bacteraemia, hyponatraemia, low mid-upper arm circumference <10 cm, hypoxia, hypokalaemia and oedema. Bacteraemia had the highest risk of death (adjusted OR 6.1; 95% C.I 2.3, 16.3 p<0.001); and complicated 24 (20%) of fatalities. Positive HIV antibody status was more frequent in cases with diarrhoea at admission (23%) than those without (15%, Χ^2^ = 12.0 p = 0.001) but did not increase the risk of death in diarrhoea cases.

**Conclusion:**

Children with SAM complicated by diarrhoea had a higher risk of death than those who did not have diarrhoea during their hospital stay. Further operational and clinical research is needed to reduce mortality in children with SAM in the given setting.

## Introduction

It is estimated that severe malnutrition accounts for 2 million deaths annually [1]. Case-fatality rates in African hospitals for severe acute malnutrition (SAM) remain unacceptably high, especially in children complicated by HIV, invasive bacterial disease or underlying medical complications [2], [3]. Whilst outcomes of cohorts of children managed for SAM in community-based therapeutic facilities and nutritional centres in nutritional emergency settings are favourable, there are important differences to children presenting to hospital in areas with high rates of endemic undernutrition. The latter are usually complicated by serious co-morbidities, such as infection, which may underpin the failure of current guidelines to improve outcome in that group [4], [5], [6].

Diarrhoea, defined as 3 or more watery stools per day, often complicates SAM [6], [7]. Management of diarrhoea and dehydration continue to be controversial issues in the treatment of severe malnutrition. In some studies, diarrhoea has been shown to predict poor outcome, especially when complicated with other features of severity [3], [8], whereas other groups have shown little impact upon successful rehabilitation [9]. We have previously shown that both community- and nosocomially-acquired diarrhoea are major challenges to the successful management of Kenyan children hospitalized with severe malnutrition, with the highest mortality occurring in the groups with clinical signs of severe dehydration and impaired perfusion [3].

Bacterial infection is more common in severe and complicated malnutrition and thought to occur in part due to the immunosuppressive effects of malnutrition and in part due to the loss of the protective mucosal barrier in the gastrointestinal tract [10]. Children with severe malnutrition have a higher prevalence of gut barrier dysfunction [7], *Escherichia coli* bacteraemia (odds ratio 4.73 (95% confidence interval 3.15–7.10) [11] and a higher mortality rate from invasive bacterial infection than well nourished children with bacteraemia (39% vs 12%) [8].

There is a lack of systematic reporting of clinical and laboratory data taken on admission to hospital to identify baseline risk factors that allow comparative studies of the burden, spectrum and outcome of severe malnutrition between hospitals. This frustrates the development of meaningful conclusions about a priori risks and the development of interventional research to target treatment priorities. Identifying risk factors and causal events underpinning these will help to inform prospective treatment studies or changes in management to improve outcome. Here we present a description of children admitted to hospital with severe acute malnutrition complicated by diarrhoea. The aim was to identify risk factors at admission in this subgroup that indicate increased risk of death during hospital stay.

## Methods

### Ethics Statement

The study was approved by the Kenyan Medical Research Institute Scientific Steering Committee and National Ethics Review Committee (protocol number 927).

The study was conducted at Kilifi District Hospital (KDH), situated on the coast of Kenya, over a period of 4 years from June 6 2005 to June 5 2009. The characteristics of the study site, population and hospital have previously been described [3], [12]. All children admitted to the ward were examined by a member of the clinical research team. Children eligible for inclusion in the study were those aged 6 months or more who had at least one feature of severe malnutrition. Severe malnutrition was defined as either: weight-for-height Z-score (WHZ, NCHS reference) <−3, or mid-upper arm circumference (MUAC) <11 cm if length over 65 cm [13] to define marasmus or kwashiorkor defined as symmetrical pitting oedema involving at least the feet to define oedematous malnutrition, excepting those with other conditions such as nephrotic syndrome, irrespective of WHZ score or MUAC [14]. The only exclusion criterion was if the caretaker declined consent. Parents or guardians of individual study participants had the study explained in their own language and gave written informed consent.

Full blood count, blood film for malaria parasites, plasma creatinine and electrolytes, plasma glucose, blood gases, blood culture and HIV rapid antibody test were collected at hospital admission. Selected stool samples were examined for reducing substances (using Clinitest tablets, Bayer, Elkhart, USA). Chest x-rays, stool microscopy and culture were requested at the discretion of the clinician. From November 2006 we implemented an opt-out system for provider initiated prospective HIV testing on admission following new recommendations by Kenyan Ministry of Health. HIV status was determined by antibody test and children with positive results were referred to the HIV Comprehensive Care and Research Clinic. Blood cultures were processed by a BACTEC 9050 system instrument (Becton Dickinson, New Jersey, USA). Antibiotic sensitivity testing of clinical isolates was performed in accordance with the British Society for Antimicrobial Chemotherapy (BSAC, UK) guidelines.

### Management

Children were treated according to WHO guidelines [7]. The multi-disciplinary study team included clinical researchers, a nurse coordinator, ward assistants, a nutritionist and a play therapist. Heart rate, respiration rate, axillary temperature, oxygen saturation (Nellcor), and presence of cold extremities were recorded every 6 hours in the first 48 hours of admission then 12 hourly. Blood glucose was also recorded 6 hourly for the first 48 hours. Children were fed 4-hourly with F75 at 130 ml/kg/day until appetite had improved and oedema had resolved, then they were changed to F100 feeds in increasing amounts up to 200 ml/kg/day as tolerated All milk feeds were freshly prepared every 4 hours according to the recipes in the WHO manual [7], in a dedicated milk kitchen by trained health staff, and included combined commercially prepared mineral and vitamin mix. Intravenous ampicillin (50 mg/kg 6 hourly) and gentamicin (7.5 mg/kg once daily) were prescribed at admission after blood cultures had been drawn. Antibiotic treatment was reassessed daily, based on clinical condition in conjunction with culture and sensitivity results. Daily clinical assessment was carried out by research medical staff and entered directly onto a computer database during the ward round. The child’s weight, type of feed (F75 or F100) and use of nasogastric feeding tube were recorded daily as well as the treatment plan for the next 24 hours. Dehydration was managed according to WHO policy. ReSoMal (Nutriset, Malauny, France) was provided for oral rehydration initially observed by nursing staff. For children too weak or unable to rehydrate orally a nasogastric tube was inserted for rehydration and feeding. Carers were encouraged to give additional ReSoMal for every loose stool. Intravenous rehydration was reserved for children with impaired consciousness, persistent vomiting, and shock (defined by the WHO in severe malnutrition as all of the following: altered conscious level, capillary refill time more than 3 seconds, a weak and fast pulse plus a temperature gradient (cool hands and/or feet)). Half strength Darrow’s solution with 5% dextrose or Ringers Lactate with 5% dextrose was prescribed and given according to WHO treatment guidelines. We administered a potassium correction (0.4 mmol/kg/hour for 4 hours) to children with plasma potassium less than 1.5 mmol/litre who had ECG changes consistent with hypokalaemia.

### Statistical Analysis

Baseline characteristics on admission were compared between those with diarrhoea (defined as 3 or more watery stools per day) and those without, to determine whether the groups differed from each other, and proportion, or median with interquartile ranges presented. Bivariate variables were created for continuous data (see Tables 1 and 2) including tachypnoea defined as 50 or more breaths per minute (brpm) if aged less than 1 year and 40 or more brpm if aged 1 year and over; and tachycardia defined as >160 beats per minute (bpm) if less than 1 year and >120 bpm if aged 1 year and over. Hypoxia was defined as oxygen saturation less than 95% on pulse oximetry. Severe dehydration was defined as 2 or more of sunken eyes or decreased skin turgour or lethargy or inability to drink.

**Table 1:** Frequency of baseline clinical features in 1206 severely malnourished children.

Admission Feature	Observationsanalyzed (n)	No diarrhoea on admissionn = 613 (% of non diarrhoea)	Diarrhoea on admissionn = 592 (% of diarrhoea)	P value
Female	1203	293(48)	280(47)	0.840
Oedema	1203	266(43)	186(31)	<0.001
MUAC <10 cm	1199	115(19)	142(24)	0.028
Hypothermia (axillary temp<35**°**C)	1205	1(0.2)	3(0.5)	0.3
Fever (axillary temp>37.5°C)	1205	265(43)	229(39)	0.110
Tachypnoea*	1188	184(30)	161(28)	0.270
Indrawing	1204	129(21)	84(14)	0.002
Hypoxia	1193	90(15)	64(11)	0.039
Bradycardia (<80 b.p. min)	1197	4(1)	5(1)	0.7
Tachycardia*	1197	176(29)	134(23)	0.014
Capillary refill time >2 seconds^a^	1204	23(4)	44(7)	0.005
Temperature gradient	1201	51(8)	74(13)	0.017
Weak pulse	1203	16(3)	46(8)	<0.001
Wasting	1202	383(63)	394(67)	0.170
Deep breathing	1204	22(4)	66(11)	<0.001
Lethargy	1203	46(8)	90(15)	<0.001
Sunken eyes	1201	48(8)	209(35)	<0.001
Reduced skin turgour	1203	47(8)	199(34)	<0.001
WHO Shock^c^	1204	0	1(0.2)	-
WHO Severe dehydration^d^	1206	37(6)	178(30)	<0.001
Impaired consciousness^b^	1203	19(3)	29(5)	0.075
Danger signs^e^	1205	139(23)	181(31)	0.002
Hyponatraemia (<125 mmol/L)	1056	26(5)	61(12)	<0.001
Hypokalaemia (<2.5 mmol/L)	1056	58(11)	182(35)	<0.001
Elevated creatinine (>80 mmol/L)	1056	45(11)	63(16)	0.039
Hypoglycaemia (<3 mmol/L)	1105	87(15)	78(15)	0.790
Acidosis (base deficit >10 mmol/L)	1018	107(20)	206(42)	<0.001
Bacteraemia	1206	38(6)	48(8)	0.200
Malaria parasitaemia	1201	126(21)	101(17)	0.120
Leucocytosis (wbc>12×10^9^/L)	1186	324(54)	329(56)	0.370
Leucopenia (wbc<4×10^9^/L)	1186	15(2)	15(3)	0.930
Severe Anaemia (hb <5 g/dL)	1186	56(9)	16(3)	<0.001
Died	1205	72(12)	122(21)	<0.001
HIV antibody positive	1017	93(15)	136 (23)	<0.001
HIV antibody not tested	1017	21(3)	46(8)	0.02

**Tables 1** **and** **2** **legend.**

a APLS definition.

b Impaired consciousness  =  prostration or coma.

c WHO definition of shock  =  impaired consciousness *plus* weak pulse volume *plus* CRT>3 seconds.

d WHO features of severe dehydration  = 2 or more of (sunken eyes or decreased skin turgour or lethargy or inability to drink).

e WHO Danger signs  =  Hypothermia or hypoglycaemia or lethargy.

**Table 2:** Clinical and laboratory features by mortality of 592 children admitted with diarrhoea.

Admission feature	Survived		Died		Odds ratio for death	95% Confidence intervals	P Value
	n = 470	%	n = 122	%			
Sex (male vs female)	216	46	64	52	0.8	0.5,1.2	0.210
MUAC<10 cm	93	20	49	41	2.8	1.8, 4.3	<0.001
Oedema	136	29	50	41	1.7	1.1, 2.6	0.009
Fever	182	39	47	39	1.0	0.7, 1.6	0.980
Tachypnoea	128	28	33	27	1.0	0.6, 1.5	0.940
Indrawing	59	13	25	20	1.8	1.1, 3.0	0.025
Hypoxia	42	9	22	18	2.3	1.3, 4.1	0.003
Deep breathing	44	9	22	18	2.1	1.2, 3.7	0.007
Temperature gradient	51	11	23	19	1.9	1.1, 3.3	0.018
Weak pulse	27	6	19	16	3.0	1.6, 5.7	<0.001
Reduced skin turgour	151	32	48	39	1.4	0.9, 2.1	0.140
Sunken eyes	154	33	55	45	1.7	1.1, 2.5	0.012
Severe dehydration	130	28	48	39	1.7	1.1, 2.6	0.012
CRT≥2 seconds	25	5	19	16	3.3	1.7, 3.3	<0.001
Lethargy	63	13	27	22	1.8	1.1, 3.0	0.017
Impaired consciousness	14	3	15	12	4.6	2.1, 9.9	<0.001
WHO Danger signs	128	27	53	43	2.1	1.4, 3.1	0.001
Hyponatraemia (<125 mmol/L)	38	9	23	21	2.6	1.5, 4.7	0.001
Hypokalaemia (<2.5 mmol/L)	128	31	54	50	2.2	1.4, 3.4	<0.001
Elevated creatinine (>80 mmol/L)	42	13	21	23	2.0	1.1, 3.6	0.021
Hypoglycaemia (<3 mmol/L)	59	14	19	17	1.3	0.7, 2.3	0.380
Acidosis (base deficit >10 mmol/L)	151	37	55	55	1.9	1.2, 3.0	0.003
Bacteraemia	24	5	24	20	4.3	2.3, 8.1	<0.001
Malaria parasitaemia	92	20	9	7	0.3	0.2, 0.7	0.001
Leucocytosis (wbc>12×10^9^/L)	256	55	73	60	1.2	0.8, 1.8	0.330
Leucopenia (wbc<4×10^9^/L)	11	2	4	3	1.4	0.4, 4.5	0.570
Severe anaemia (hb <5 g/dL)	14	3	2	2	0.5	0.1, 2.4	0.410
HIV Antibody status Negative	308	65	67	55	1		
Positive	107	23	29	24	1.2	0.8, 2.0	0.38
Not tested	55	12	26	21	2.2	1.3, 3.7	0.0042

The admission features were analysed to look for an association with outcome among only those children with diarrhoea and crude odds ratios for death with 95% confidence intervals were obtained. Logistic regression analysis was used to determine predictors of death in these children. Predictors that were significant at the 10% level in the univariable analyses were considered for inclusion in the multivariable regression model. HIV antibody status was included in the model together with a dichotomous variable for age, with a cutoff at 18 months, the age below which positive serology may be due to exposure, with passive transfer of maternal antibody, rather than infection. It was expected that there would be marked collinearity between many of the variables for clinical signs associated with poor perfusion and dehydration. For variables found to exhibit collinearity with each other, a single variable was chosen from them for inclusion in the multivariable analysis. All statistical analyses were conducted in Stata version 10 (StataCorp LP, College Station, Texas, USA).

## Results

In a 4-year period there were 12,857 paediatric admissions aged 6 months and above, of whom 1,628 (13%) fulfilled the anthropometric criteria for severe malnutrition, and 1,206 (74% of these, 9% of all admissions) were recruited to the study. Fourteen parents refused consent (1% of those fulfilling entry criteria) and 28 (2%) did not come with a guardian who could act as a proxy for consent. The remaining 360 children were not enrolled either because they were readmissions with severe malnutrition, their eligibility was not picked up at admission or they were suspected to have an alternative primary diagnosis (non-malnutrition cause of oedema).

### General Characteristics

Overall, the median age was 22 months [interquartile range 15 to 34], 91% of children were less than 5 years old and 52% were male. Oedematous malnutrition constituted 452 (38%) of the cases. HIV status was available for 1017 (84%) of study patients: 229 (19% of 1,206) were antibody positive, of these 159 (16%) were aged 18 months or more (HIV infected) and 70 (7%) were aged less than 18 months and were considered as HIV exposed. Overall, 194 (16%) children died during their hospital stay. Twenty two of these deaths (11%) occurred within 48 hours of admission. The children who were not recruited but fulfilled the admission anthropometric criteria for the study had a median age of 27 months (IQR 17 to 50) with a case fatality of 15%.

Within this cohort (n = 1,206), five hundred and ninety two children (49%) were admitted with a history of diarrhoea. The median duration of diarrhoea before admission was 5 days (IQR 3, 14 days) and of those with diarrhoea only 86 (15%) children had chronic diarrhoea persisting for more than 14 days. Oedema was less prevalent in cases with diarrhoea than non-diarrhoeal cases (see Table 1) Clinical features of impaired perfusion and acidotic breathing, as well as signs of severe dehydration, were more common in children admitted with diarrhoea than non-diarrhoeal cases (Table 1). Of the WHO danger signs, lethargy was more prevalent in diarrhoea cases (15%) than non-diarrhoea cases (8%) whereas hypothermia or the strict WHO definition of shock were rare (<0.5%) in all children. Hypoglycaemia at admission (blood glucose <3.0 mmol/l) was equally common in the two groups.

### Outcome

One hundred and twenty two of the children admitted with diarrhoea died (21%) compared to 72/614 (12%) without diarrhoea on admission (Χ^2^ = 17.6 p<0.001). A further 187 (16%) children admitted without diarrhoea developed diarrhoea (> than 3 loose watery motions) more than 48 hours after admission of whom 33/187 died (18%). The mortality for children who had diarrhoea at any time during their admission 161/852 (19%) was significantly higher than those whose hospital stay was not complicated by diarrhoea 33/351 (9%) (Χ^2^ = 16.6 p<0.001).

**Figure 1:**
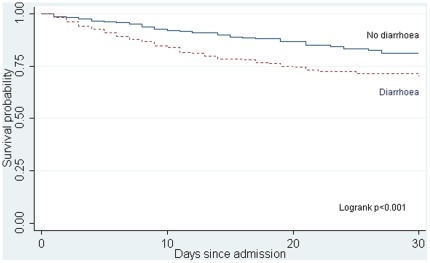
Kaplan-Meier plot of survival time of children with SAM admitted with and without diarrhoea.

The timing of death differed between diarrhoea and non-diarrhoea groups (Figure 1) log-rank p-value<0.001.

### Risk Factors for Mortality

Admission characteristics were compared between survivors and fatal cases in children admitted with a history of diarrhoea (Table 2). One hundred and seventy eight children (30%) admitted with a history of diarrhoea had signs of severe dehydration. Severe dehydration was associated with a higher risk of death (crude odds ratio 1.7; 95% CI 1.1, 2.6; p = 0.012), Markers of impaired perfusion (including any one of delayed capillary refill 3 or more seconds, temperature gradient or weak pulse volume), hyponatraemia, hypokalaemia or high creatinine (a marker of renal perfusion) were more common in fatal cases and increased the risk of death (see Table 2). Of the WHO malnutrition-specific danger signs (lethargy, hypothermia or hypoglycaemia), only lethargy was discriminatory for outcome.

Bacteraemia, detected by admission blood culture, was a major risk factor for death in those admitted with diarrhoea (crude Odds Ratio (OR)  = 4.3; 95% C.I 2.3, 8.1 p<0.001); of all the fatalities 24/122 (20%) had a positive admission blood culture. For children admitted with diarrhoea there was no difference in the crude odds ratio of dying associated with HIV status (O.R  = 1.2 95% C.I 0.8, 2.0 p = 0.38) among those tested; however those not tested were at a higher risk of dying (OR 2.2 95% C.I 1.3, 3.7 p = 0.004).

The development of signs of shock indicating the need for intravenous rehydration occurred in 156 (26%) children who had diarrhoea. This group was characterised by a high prevalence of hypokalaemia (<2.5 mmol/L 90 (58%)), raised creatinine (>80 mmol/L 55 (35%)) and severe acidosis (base excess<−10 mmol/L 49 (31%)). Outcome was poor: 68 (44%) died.

### Multivariable Analysis

The variables significantly associated with death in the multivariable analysis are shown in Table 3. Children with bacteraemia or hyponatraemia had the greatest odds of dying (adjusted OR 6.1 95%CI 2.3, 16.3 P = <0.001 and 4.6 95%CI 2.0, 10.6 P<0.001 respectively). Other predictors of death in children admitted with diarrhoea included having a MUAC below 10 cm, hypoxia, hypokalaemia and oedema. The variables for HIV serology and age were displaced from the final model during the regression analysis.

**Table 3:** Multivariable logistic regression variables associated with death in children with severe malnutrition and diarrhoea at admission.

		Model including MUAC		Model excluding MUAC		
Variable	Odds Ratio	95% Confidence Interval	P value	Odds Ratio	95% Confidence Interval	P value
Bacteraemia	6.1	2.3, 16.3	<0.001	6.7	2.5,17.8	<0.001
Hyponatraemia	4.6	2.0, 10.6	<0.001	4.9	2.2, 11.1	<0.001
MUAC <10 cm	3.5	1.9, 6.5	<0.001			
Hypoxia	2.6	1.1, 6.1	0.033	2.5	1.1,5.8	0.03
Hypokalaemia	2.5	1.3, 4.6	0.004	2.3	1.3, 4.2	0.007
Oedema	2.1	1.1, 4.0	0.019	1.9	1.0,3.4	0.045

Different models with and without low MUAC were tested because MUAC has been reported to vary with hydration status.

### Bacterial Isolates and Reducing Substances

Admission blood culture isolates amongst children admitted with SAM and diarrhoea are shown in Table 4. The most common organisms were *Streptococcus pneumoniae* (27%), *Escherichia.coli* (19%) and *Salmonella* species (15%). Blood cultures taken later on in the hospital stay (n = 240) were positive in 23/592 children with diarrhoea at admission compared to 12/614 children in those without (Χ^2^ = 4.0 p = 0.05). Post-admission isolates (nosocomial) were predominantly Gram-negative (83%) compared to only 54% of admission blood culture isolates. The main organisms responsible for nosocomial-acquired infection were *E.coli* (39%), *Klebsiella* species (22%) and *Pseudomonas* species (17%).

**Table 4:** Blood culture isolates in children admitted with diarrhoea.

Organism	AdmissionIsolates N (%)	Post-admissionIsolates N (%)
Gram negative		
*Escherichia coli*	9 (19)	9 (39)
*Salmonella* spp	7 (15)	–
*Klebsiella* spp	2 (4)	5 (22)
*Haemophilus influenzae*	2 (4)	–
*Acinetobacter* spp	3 (6)	–
*Pseudomonas* spp	1 (2)	4(17)
*Aeromonas hydrophila*	1 (2)	–
*Morganella morganii*	1 (2)	–
*Proteus mirabilis*	–	1 (4)
**Subtotal**	**26 (54)**	**19 (83)**
**Gram positive**		
*Streptococcus pneumoniae*	13 (27)	–
*Staphylococcus aureus*	4 (8)	1(4)
*Streptococcus* group A	1 (2)	–
*Streptococcus* group D	2 (4)	3 (13)
*Streptococcus* group G	2 (4)	–
**Subtotal**	**22 (46)**	**4 (17)**
**Total**	**48 (100)**	**23 (100)**

In vitro testing of admission blood culture isolates from patients admitted with diarrhoea against the standard WHO recommended antibiotic combination of ampicillin and gentamicin demonstrated that 67% (32/48) were sensitive to first line antibiotics; 90% were sensitive to the second line antibiotic chloramphenicol; 71% of isolates were sensitive to ceftriaxone and 80% to ciprofloxacin. In 48 children with bacteraemia and diarrhoea at admission, 24 died (50%). Isolates from post-admission blood cultures were less sensitive to commonly used antibiotics: of the 23 tested from patients admitted with diarrhoea only 35% were sensitive to the ampicillin and gentamicin combination, 37% to chloramphenicol, 32% to ceftriaxone and 44% to ciprofloxacin. Mortality was high in this group: 14/23 (61%).

Stool culture was not routinely performed, but was requested in 151 cases due to bloody diarrhoea, high-output severe diarrhoea or refractory diarrhoea (29 were repeated stool cultures). Pathogens were only cultured in 7 specimens from 7 children; *Shigella flexneri* 4, *Salmonella* 2 and *Campylobacter* 1. Stools were tested for reducing substances in 111 of the diarrhoea cases on the day of admission, usually before milk feeding. These were positive in 8% (9/111) of which 2 children died.

## Discussion

We have shown that diarrhoea is a common complication (49%) in children hospitalised with severe acute malnutrition. Overall, mortality for children who had diarrhoea at any time during their admission was significantly higher (19%) than the mortality without diarrhoea (9%). Many children who were admitted with diarrhoea had concurrent features of severe dehydration, impaired perfusion or severe metabolic acidosis- mortality in this group was substantial. Of note was that only one patient at admission met the strict WHO shock criteria and thus eligible for fluid resuscitation. Bacteraemia and hyponatraemia were the factors associated with the highest risk of death, irrespective of HIV or MUAC status.

Our cohort had a lower mortality in the first 48 hours of admission (11%) than the cohort of severely malnourished children admitted to our hospital between September 2000 and June 2002 (33%,Χ^2^ = 26.6 p<0.001) previously described. [3] This may have resulted from improved emergency triage and early supportive treatment, however mortality during hospital stay (16%) was similar to the earlier cohort (19%, Χ^2^ = 3.4, p = 0.07).

Stool culture data from a small proportion (13%) did not allow us to describe the population prevalence of pathogenic diarrhoea; however the results in those children with stool microbiological data suggested that a primary bacterial aetiology for diarrhoea was infrequent, unlike cohorts described in Bangladesh [15], [16] where *Vibrio cholerae* and *Shigella* species were common. The possibility exists that delays in sample transfer and processing may have resulted in a low isolation rate of pathogens. More likely explanations are pre-hospital use of antibiotics or associated enteropathy which has been described in other populations [17]. Concomitant bacteraemia was common (7%) in all children admitted with malnutrition but had no particular association with diarrhoea at admission. However, bacteraemia even on appropriate antimicrobial treatment, remained the greatest independent risk factor for death in those admitted with diarrhoea (adjusted OR  = 6.1; 95% C.I 2.3, 16.3). Although bacteraemia was less common in our unselected cohort of hospitalized severely malnourished children than that described by Bachou in Kampala, Uganda (17%), in both studies the proportions of Gram-negative and Gram-positive organisms were similar [18].

The antimicrobial sensitivities of admission blood culture isolates to the first line combination of ampicillin and gentamicin were only 67%. This proportion was similar to findings of a previous study examining antimicrobial sensitivities of invasive gram negative bacterial isolates in children admitted to our hospital between 1994 to 2001 showing that 73% were sensitive to gentamicin [19]. In both studies we found no effect of resistance on mortality. In the current study non-typhoidal *Salmonellae* (NTS) were the second most common Gram-negative blood culture isolates. Gentamicin, although not indicated by sensitivity testing, is not recommended for treatment of NTS since it is poorly active against such intracellular organisms. For all Gram-negative organisms sensitivities were greatest to ciprofloxacin. We have previously shown that the bioavailability and pharmacokinetics of a 30 mg/kg/day oral preparation of this drug in severely malnourished children indicates adequate (>80%) cumulative fraction of response for *E. coli, Klebsiella pneumoniae* and Salmonella species, but poor (60%) for *Pseudomonas aeruginosa*. [20], [21]. Prognosis was extremely poor in cases complicated by hospital-acquired bacteraemia; case fatality was 61%, emphasizing the importance of effective hospital infection control measures for severely malnourished children [22]. Children with severe malnutrition complicated by diarrhoea are cohorted in a dedicated area of the ward. Medical and nursing staff undergo regular training on handwashing and prevention of cross-infection.

We suspected that impaired gut barrier function may be prevalent in our population with severe malnutrition owing to the poor outcome in those with diarrhoea and the development of purging diarrhoea on nutritional rehabilitation. Although not systematically tested, reducing substances (indicating disaccharide intolerance), were only present in 8% of stools taken at admission but this usually preceded a lactose challenge, thus the true prevalence was likely to be underestimated. In malnourished Ugandan children higher mean stool frequency was found to be an independent predictor of lactose intolerance [23]. In Malawian children with kwashiorkor there was a higher prevalence of gut barrier dysfunction, defined by decreased absorption of L-rhamnose and increased permeation of lactulose in cases with diarrhoea and fatal outcome [10]. In Australian Aboriginal children who were underweight, osmotic diarrhoea was a common complication and linked to lactose intolerance and increased intestinal permeability leading to severe purging, hypokalaemia and metabolic acidosis [24]. In these children a low osmolarity, low lactose milk formula resulted in significantly better weight gain than either a low lactose or partially hydrolysed milk formula (both having higher osmolarities) [25]. A Zambian controlled trial of 4 weeks’ feeding with an amino acid-based elemental feed in severely malnourished or underweight children with persistent diarrhoea found better weight gains and greater increases in haemoglobin level than in children fed on cow’s milk or soya based formulas [26]. Taken together there appears to be mounting evidence to support the adverse outcome in children with SAM complicated by diarrhoea. Impaired brush barrier function of the small intestine may be more common than previously considered - indicated by lactose intolerance and other markers of impaired permeability in previous studies and the increased susceptibility to Gram-negative bacteraemia in our study. We hypothesise that the osmolarity and lactose content of the milk-based feeds are important factors underpinning the development of severe, high output, osmotic diarrhoea with life-threatening consequences and prolongation of rate of recovery. The osmolarity of F75 and F100 milk formulas, including both commercially-produced and milk formulae prepared locally in accordance to standard recommendations, were measured serially over one year and were found to be hyperosmolar (means 333 and 550 milliosmoles/litre respectively).

One of the greatest challenges to successful treatment of malnourished children with diarrhoea was the management of severe dehydration and/or shock. Under the current guidelines for severely malnourished children, dehydration is managed on oral replacement with low sodium rehydration solution (ReSoMal). Intravenous fluid resuscitation is reserved for children with advanced shock (impaired consciousness plus three signs of impaired perfusion) only and with no consideration for rehydration. The high mortality of children managed on WHO recommendations and the prominence of severe metabolic acidosis, raised creatinine, severe hyponatraemia and hypokalaemia are of substantial concern and raised questions over whether these recommendations should include intravenous rehydration. A trial in severely malnourished Bangladesh children evaluating the safety and efficacy of oral rehydration with ReSoMal compared to standard WHO-ORS noted an equal efficacy but more children in the ReSoMal group remained hyponatraemic after 24 hours of rehydration (29% vs 10% p = 0.017) and 3 of these children developed symptomatic severe hyponatraemia including one experiencing convulsions [27]. Nevertheless, after that trial ReSoMal was adopted into current management guidelines on the grounds of superior potassium correction. Intravenous replacement of deficits has been examined in two trials. The first was conducted in Bangladeshi children with severe malnutrition complicated by cholera and demonstrated the safety of up to 100 ml/kg of isotonic fluid given over 6 hours and low mortality. However, cholera represents a special case, with disproportionately large fluid losses, which may not be generalizable to other cohorts with diarrhoea [15]. In a recent trial, comparing isotonic fluid resuscitation to standard of care (WHO recommended half-strength Ringers lactate/5% Dextrose solution) in children with severe malnutrition and hypovolaemia, mortality in the diarrhoeal group managed on WHO protocol was 68% (13/19) compared to 43% (9/22) in the arm receiving equivalent volumes of isotonic Ringers Lactate (p = 0.11) [28]. There were no reports of cardiac failure, pulmonary oedema or fluid overload. Nevertheless, management of shock in children irrespective of nutritional status remains controversial. In non-malnourished children with shock, a large pragmatic fluid resuscitation trial (FEAST) recently concluded showing a higher mortality in children managed with boluses compared to non- bolus [29]. In children with severe malnutrition complicated by diarrhoea there is an urgent need to examine in well-powered trials the efficacy and effectiveness of a bundle of care (including strategies of oral and intravenous rehydration and integration with feeding) to improve the currently poor outcome.

In conclusion, at our hospital almost half of children admitted with severe acute malnutrition were complicated by diarrhoea and these children were at increased risk for death. Frequent monitoring for signs associated with clinical deterioration should be carried out in these children. Further research is needed into therapeutic strategies to treat the gut dysfunction associated with malnutrition.
